# Foveal reorganization after treatment of acute foveal toxoplasmic retinochoroiditis

**DOI:** 10.1186/s12348-021-00246-2

**Published:** 2021-06-21

**Authors:** Mojtaba Abrishami, Seyedeh Maryam Hosseini, Solmaz Momtahen, Ghodsieh Zamani

**Affiliations:** Eye Research Center, Khatam-al-Anbia Eye Hospital, Mashhad University of Medical Sciences, Qarani Blvd, Mashhad, 9195965919 Iran

**Keywords:** Toxoplasmic Retinochoroiditis, Clindamycin, Intraocular injection, Fovea

## Abstract

**Purpose:**

To report a patient with impaired vision due to foveal involvement of toxoplasmic retinochoroiditis, who was successfully treated with intravitreal clindamycin and dexamethasone and oral therapy with azithromycin, trimethoprim-sulfamethoxazole, and prednisolone and led to successful visual and anatomic recovery.

**Case presentation:**

A 32-year-old man presented with three-day history of gradually decreasing visual acuity, redness, pain and photophobia of the right eye. Anterior chamber cellular reaction, vitritis and a white retinochoroiditis patch with adjacent retinal vasculitis in the fovea was suggestive of the toxoplasmic retinochoroiditis. He was treated with intravitreal clindamycin and dexamethasone injection followed by six-week regimen of azithromycin, trimethoprim-sulfamethoxazole, and prednisolone. In serial optical coherence tomography imaging, retinitis patch changed to cavitary foveal destruction. Fovea reorganized gradually, and visual acuity concurrently improved from counting finger 3 m to 20/25.

**Conclusion:**

In foveal toxoplasmic retinochoroiditis lesions, timely treatment is associated with retinal reorganization and visual improvement.

## Introduction

Toxoplasmic retinochoroiditis caused by Toxoplasma gondii is a potential cause of posterior uveitis that could lead to blindness. It occurs in the posterior pole in more than 50% of cases [1]. Symptoms of ocular toxoplasmosis usually include a unilateral decrease in vision with floaters, accompanied by signs of anterior uveitis, and around one fifth of patients have increased intraocular pressure [2]. In the posterior segment, the condition classically appears as focal, grayish-white retinitis with overlying moderate vitreous inflammation, often adjacent to a pigmented chorioretinal scar [2]. The diagnosis of toxoplasma retinochoroiditis is clinical in most instances. Visual loss caused by ocular toxoplasmosis arises from complications due to structural changes and the effects of associated intraocular inflammation and scar formation [2]. As the focal lesion of necrosis of the retina and choroid heals, a permanent punched-out chorioretinal scar will develop. Due to the high prevalence of the lesion occurring at the posterior pole, it is considered as a cause of severe vision loss [2]. The inflammation process itself may create inadvertent complications that can lead to permanent conditions if left unattended in a timely manner [3].

Here we report a patient with impaired vision due to foveal involvement of toxoplasmic retinochoroiditis, who was successfully treated with intravitreal antibiotic and corticosteroid injection followed by six-week oral therapy and had near complete visual recovery.

## Case presentation

**Fig. 1:**
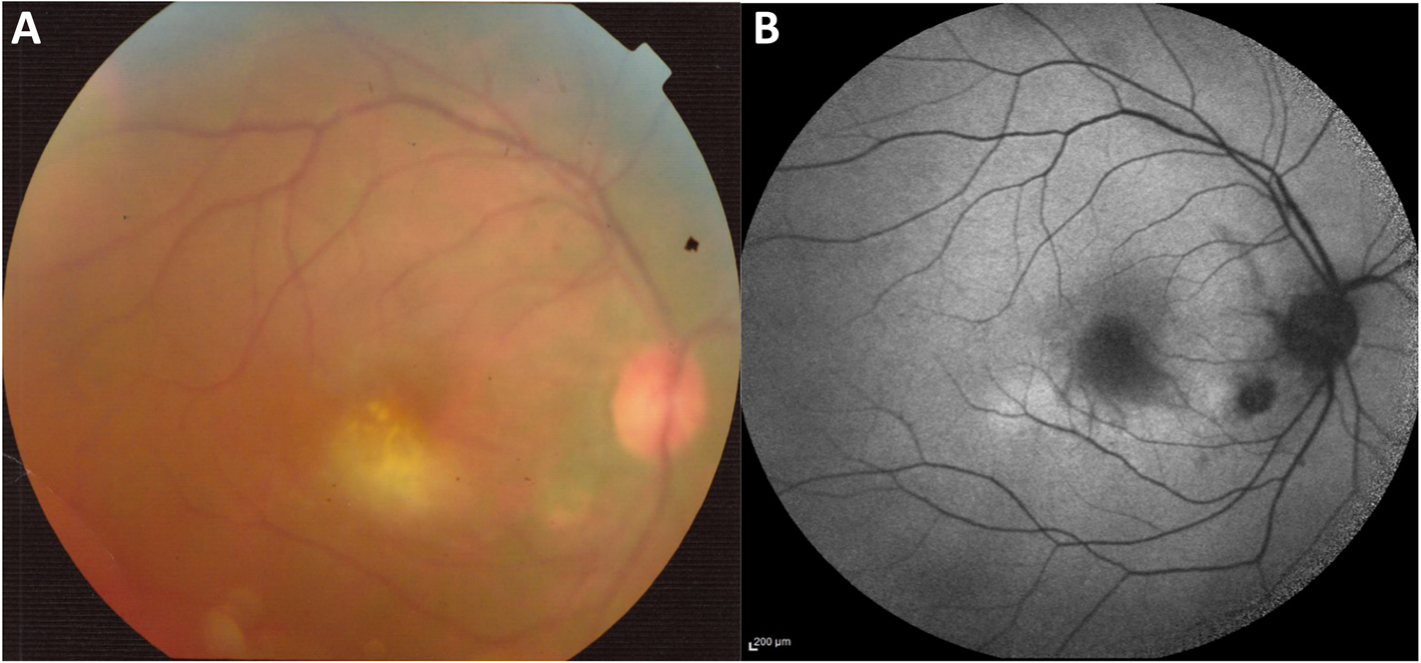
**a** Color fundus photograph of the right eye  at presentation. Media haziness and white retinochoroiditis patch about one-disc diameter in size in the foveal area. **b** Fundus autofluorescence of the right eye  at presentation

**Fig. 2:**
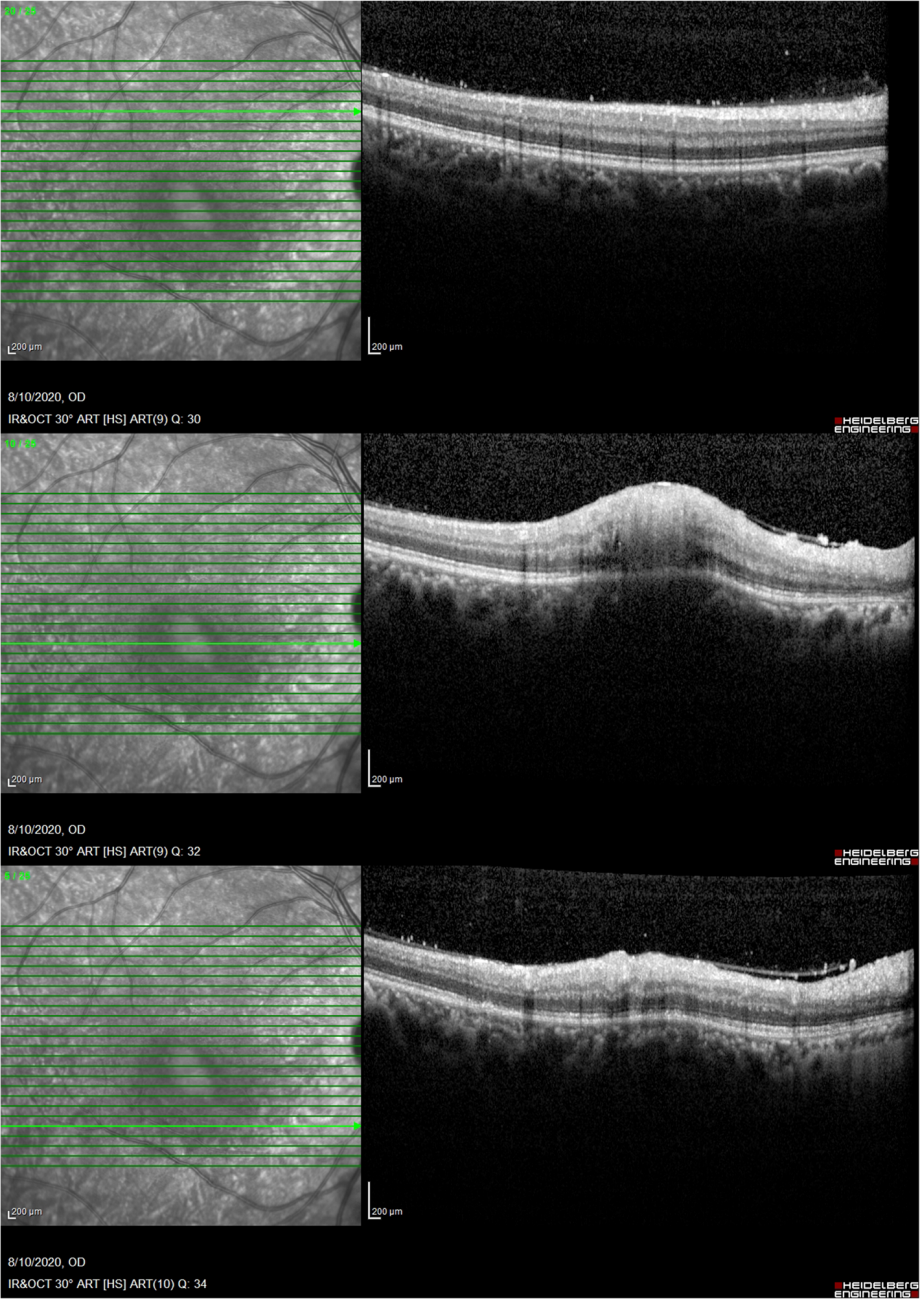
OCT of the right eye  at presentation. Vitreous inflammation, cell clumps on the retinal surface, focal hyperreflectivity of retinal layers, retinal thickening and edema

A 32-year-old man presented with three-day history of decreased visual acuity, redness, pain and photophobia of the right eye. Best corrected visual acuity (BCVA) was counting finger 3 m in the right eye and 20/20 in the left eye. Relative afferent pupilary defect was absent. Intraocular pressure was within normal limits. In slit lamp biomicroscopy of the right eye, conjunctival injection and 3+ cell and 2+ flare in anterior chamber was found. Lids, cornea and lens were normal. Fundus examination revealed vitritis, media haziness, and a round, fluffy, white retinochoroiditis patch, about one-disc diameter in size, in the foveal area associated with edema and retinal vasculitis. (Fig. 1) Left eye examination was normal. These findings were compatible with the diagnosis of ocular toxoplasmic retinochoroiditis in the active phase. The clinical suspicion of toxoplasmosis was confirmed by positive serology tests of anti-toxoplasma IgG and IgM antibodies. Foveal involvement was documented with optical coherence tomography (OCT). At first, OCT images showed foveal retinal thickening and edema (Fig. 2) and in enhanced-depth imaging (EDI-OCT) choroidal thickening was obvious. Because of the proximity of the lesion to the fovea, treatment was initiated immediately with intravitreal injection of dexamethasone (0.4 mg/0.1 mL) and clindamycin (1.0 mg/0.1 mL) in the right eye. It was followed by oral azithromycin (250 mg/day) and trimethoprim-sulfamethoxazole (160 mg/800 mg twice daily), topical betamethasone (every 4 hours) and homatropine (twice a day) for up to 6 weeks. Oral prednisolone (50 mg/day) was added to the above regimen from the fourth day of treatment.

**Fig. 3:**
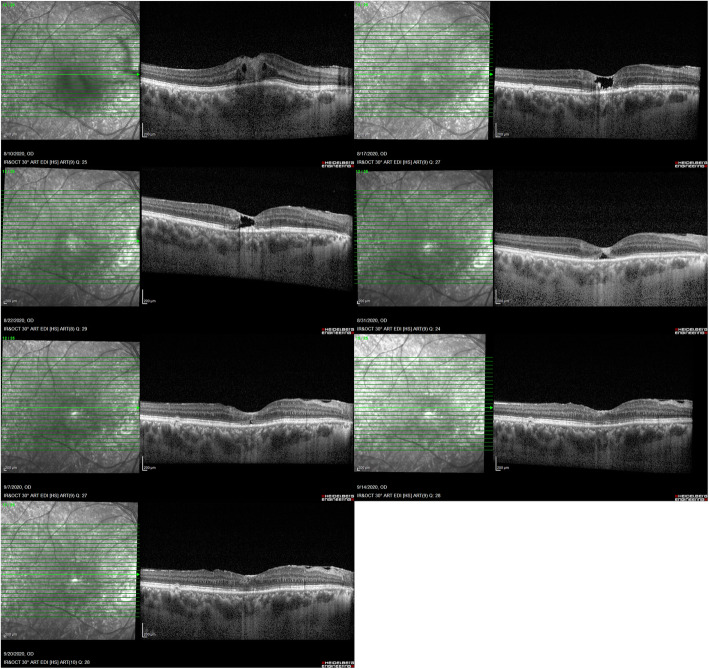
Serial EDI-OCT of the right eye (raster scans at the level of foveal center: 12/25). Progressive changes in the fovea including edema, cystic changes, subsiding edema and finally reorganization of retinal layers and foveal pit

**Fig. 4:**
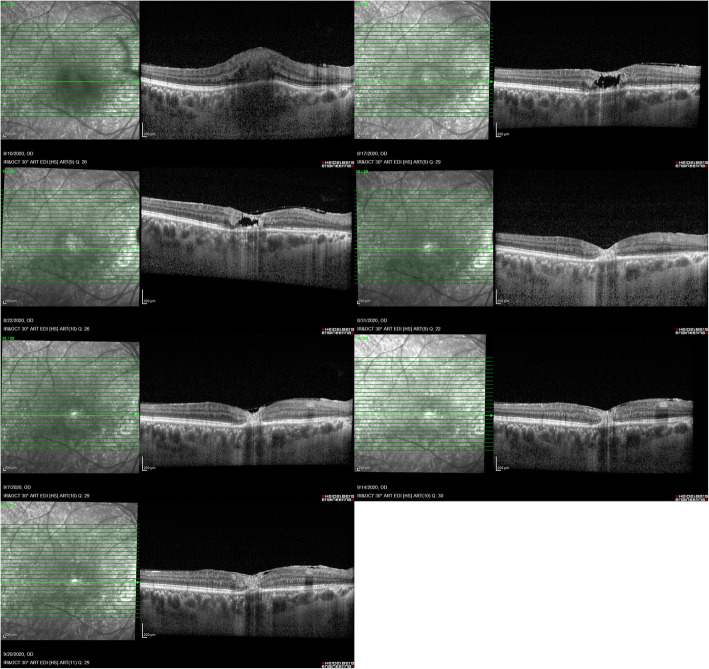
Serial EDI-OCT of the right eye (raster scans at the level of lesion center: 10/25). Progressive changes including edema, cystic changes, subsiding edema and finally scar formation at the lesion center

**Fig. 5:**
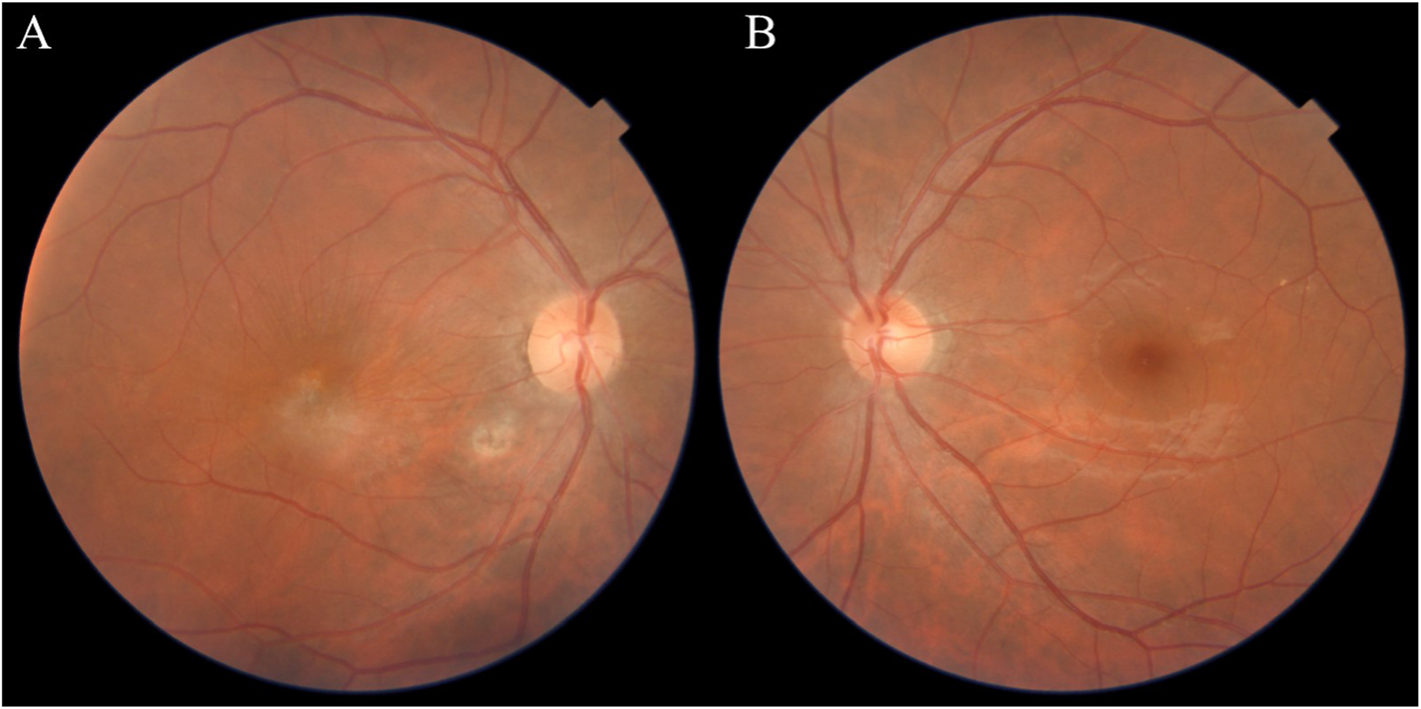
Color fundus photograph of both eyes after treatment

One week after starting the treatment, retinal and choroidal thickening subsided and cavitary retinal changes attributable to retinal destruction developed in EDI-OCT.  When comparing the follow up OCT raster scans at the same level, we noticed that fovea was reorganized gradually and foveal retinal layers integrity which had been lost, returned progressively (Figs. 3 and 4). After 6 weeks of treatment, vitritis was resolved and edema around the lesion resorbed completely (Fig. 5). Simultaneously with these anatomical changes, the right eye vision improved significantly to 20 /25.

## Discussion

Herein, we report a patient with foveal toxoplasmic retinochoroiditis that showed anatomical reorganization of the fovea with intravitreal and oral antibiotics and corticosteroid. Response to treatment and control of infection and inflammation was associated with the reformation of the foveal pit and restoration of the layered structure of the tissue. While macular toxoplasmic retinochoroiditis are usually associated with scar formation and severe visual reduction, our patient’s visual acuity was well recovered, most probably due to foveal reorganization.

Different modes of foveal regeneration were proposed in spontaneous or surgical closure of macular hole (MH): 1) regular regeneration resulted in the formation of a fovea which contained a foveola and photoreceptors in the center; 2) irregular regeneration resulted in the formation of a fovea which did not contain a foveola and central photoreceptors and was filled by a tissue formed by Müller and retinal pigment epithelial cells [4]. Because the fovea is free of astrocytes [5], the closure is likely mediated by Müller cells. Concentric contraction of the Müller cell processes envelops the photoreceptor cells in the outer nuclear layer and at the external limiting membrane and result in the centripetal movement of photoreceptor cells [4]. The irregular regeneration was mediated by proliferation of Müller and retinal pigment epithelial cells [6, 7]. It was suggested that closure of small MHs and the subsequent reconstruction of the normal foveal structure are mediated by active mechanisms of Müller cells, without cell proliferation, that resemble those involved in ontogenetic foveal development [7]. We believe that reorganization of fovea in our patient may be related to Müller cells, like what happens in MH repair.

Histopathologic findings in an eye with active toxoplasmic retinochoroiditis show cell infiltration and edema, initially in the internal retina and then in the vitreous and choroid. As a result of the infiltration, there is a disorganization of the retinal layers [8, 9]. This hystopathological feature can be correlated to the spectral domain optical coherence tomography (SD-OCT) findings of hyper-reflectivity and increased thickness of retina at the lesion site [10]. Some of the SD-OCT findings in reactivation of ocular toxoplasmosis have been described previously which included increased reflectivity in the inner retina, shadowing of the outer retina layers, thickened and detached posterior hyaloid with irregular hyperreflective formations [11–13]. Alwassia A. et al. [14] described the progression of retinitis in a case with acute ocular toxoplasmosis using SD-OCT. In this patient, the development of cystic spaces in the area of previous hyperreflectivity possibly represents the progression from retinitis to liquefactive necrosis as a result of inflammation. The retina in this case began a healing process as the gap within the retina caused by necrosis became smaller. Our patient also initially underwent cavitary changes, similar to the evolution of a MH, possibly due to a liquefactive necrosis. MH formation has been previously described as a complication of ocular toxoplasmosis [15–18]. Its pathogenesis in association with toxoplasmic retinochoroiditis is controversial, although vitreomacular traction caused by inflammation of the vitreous, changes in the posterior hyaloid membrane and posterior vitreous detachment [15, 16] and/or retinochoroidal ischemia based on the presence of retinochoroidal hypoperfusion [17] have been speculated. Tanaka R. et al. [19] presented a case of ocular toxoplasmosis with the development of a giant MH during treatment for posterior uveitis which was not closed despite vitrectomy. In another report, Doshi S. et al. [20] presented a young male with the diagnosis of left eye toxoplasma retinochoroiditis who was treated with oral co-trimoxazole and prednisolone and a single dose of intravitreal clindamycin along with dexamethasone. After 1 week, the vision dropped with the fundus showing resolving retinitis and a full thickness MH confirmed on OCT scan. One week following the occurrence of the MH, though the vision remained stable, there was spontaneous closure of the MH. They hypothesized that a limited separation of the posterior hyaloid that there was above the fovea after the intravitreal injection may be the reason for development of the MH. The strong vitreoretinal adhesion at the site of active retinitis might have prevented this hyaloid detachment from progressing, further causing its collapse and formation of a scaffold over which the MH closed. Fortunately, in our patient the healing process began with fluid absorption and the changes did not progress to the MH formation.

In conclusion, we believe that although toxoplasmic retinochoroiditis lesions often heal with atrophy and scar, early treatment may prevent scar formation and allows retinal layer reorganization.

## Data Availability

The datasets used and/or analyzed during the current study are available from the corresponding author on reasonable request.

## Abbreviations

BCVA: Best corrected visual acuity
EDI-OCT: Enhanced depth imaging optical coherent tomography
SD-OCT: Spectral domain optical coherence tomography
MH: Macular hole

## References

[1] Balasundaram MB, Andavar R, Palaniswamy M, Venkatapathy N (2010). Outbreak of acquired ocular toxoplasmosis involving 248 patients. Arch Ophthalmol.

[2] Butler NJ, Furtado JM, Winthrop KL, Smith JR (2013). Ocular toxoplasmosis II: clinical features, pathology and management. Clin Exp Ophthalmol.

[3] Cordeiro CA, Vieira EL, Castro VM (2017). T cell immunoregulation in active ocular toxoplasmosis. Immunol Lett.

[4] Bringmann A, Jochmann C, Unterlauft JD, Wiedemann R, Rehak M, Wiedemann P (2020). Different modes of foveal regeneration after closure of full-thickness macular holes by (re) vitrectomy and autologous platelet concentrate. Int J Ophthalmol.

[5] Bringmann A, Syrbe S, Görner K, Kacza J, Francke M, Wiedemann P, Reichenbach A (2018). The primate fovea: structure, function and development. Prog Retin Eye Res.

[6] Chung H, Byeon SH (2017). New insights into the pathoanatomy of macular holes based on features of optical coherence tomography. Surv Ophthalmol.

[7] Bringmann A, Duncker T, Jochmann C, Barth T, Duncker GIW, Wiedemann P (2020). Spontaneous closure of small full-thickness macular holes: presumed role of Müller cells. Acta Ophthalmol.

[8] Frenkel J, Jacobs I (1958). Ocular toxoplasmosis: pathology, diagnosis and treatment. Arch Opthalmol.

[9] Gallagher MJ, Yilmaz T, Cervantes-Castaneda RA, Foster CS (2007). The characteristic features of optical coherence tomography in posterior uveitis. Br J Ophthalmol.

[10] Garg S, Mets MB, Bearelly S, Mets R (2009). Imaging of congenital toxoplasmosis macular scars with optical coherence tomography. Retina..

[11] Cho DY, Nam W (2012). A case of ocular toxoplasmosis imaged with spectral domain optical coherence tomography. Korean J Ophthalmol.

[12] Oréfice JL, Costa RA, Campos W, Calucci D, Scott IU, Oréfice F (2006). Third-generation optical coherence tomography findings in punctate retinal toxoplasmosis. Am J Ophthalmol.

[13] Oréfice JL, Costa RA, Oréfice F (2007). Vitreoretinal morphology in active ocular toxoplasmosis: a prospective study by optical coherence tomography. Br J Ophthalmol.

[14] Alwassia A, Cho H, Adhi M, Duker JS, Baumal CR (2013). Sequential optical coherence tomography images of retinal necrosis in acute ocular toxoplasmosis. Retin Cases Brief Rep.

[15] Blaise P, Comhaire Y, Rakic JM (2005). Giant macular hole as an atypical consequence of a toxoplasmic chorioretinitis. Arch Ophthalmol.

[16] Arana B, Fonollosa A, Artaraz J, Martinez-Berriotxoa A, MartinezAlday N (2014). Macular hole secondary to toxoplasmic retinochoroiditis. Int Ophthalmol.

[17] Panos GD, Papageorgiou E, Kozeis N, Gatzioufas Z (2013) Macular hole formation after toxoplasmic retinochoroiditis. BMJ Case Rep 2013:bcr2013008915. 10.1136/bcr-2013-008915. 10.1136/bcr-2013-008915 PMC3618842 23470676

[18] Atmaca LS, Simsek T, Batioglu F (2004). Clinical features and prognosis in ocular toxoplasmosis. Jpn J Ophthalmol.

[19] Tanaka R, Obata R, Sawamura H, Ohtomo K, Kaburaki T (2014). Temporal changes in a giant macular hole formed secondary to toxoplasmic retinochoroiditis. Can J Ophthalmol.

[20] Doshi S, Gulati M, Pathengay A, Hegde S (2020). Spontaneous closure of macular hole in a case of toxoplasma retinochoroiditis. Indian J Ophthalmol.

